# Negative Perception of Aging Is Associated With Frailty Transitions Within a Cohort of Sexual Minority Men

**DOI:** 10.1093/geroni/igab035

**Published:** 2021-09-03

**Authors:** Karen Nieves-Lugo, Deanna Ware, Keri Althoff, Mark Brennan-Ing, Steven Meanley, Andre L Brown, Sabina A Haberlen, Mary Masters, James E Egan, Mackey R Friedman, Michael Plankey

**Affiliations:** 1 Department of Psychology, The George Washington University, Washington, District of Columbia, USA; 2 Department of Medicine, Georgetown University Medical Center, Washington, District of Columbia, USA; 3 Department of Epidemiology, Johns Hopkins Bloomberg School of Public Health, Baltimore, Maryland, USA; 4 Brookdale Center for Healthy Aging, Hunter College, City University of New York, New York City, New York, USA; 5 Department of Family and Community Health, University of Pennsylvania School of Nursing, Philadelphia, Pennsylvania, USA; 6 Department of Behavioral and Community Health Sciences, Graduate School of Public Health, University of Pittsburgh, Pittsburgh, Pennsylvania, USA; 7 Department of Medicine, Division of Infectious Diseases, Northwestern University Feinberg School of Medicine, Chicago, Illinois, USA; 8 Department of Infectious Diseases and Microbiology and Center for LGBT Health Research, Graduate School of Public Health, University of Pittsburgh, Pittsburgh, Pennsylvania, USA

**Keywords:** Attitudes and perception toward aging/aged, HIV/AIDS, Quantitative research methods, Sexual minority men

## Abstract

**Background and Objectives:**

Older people have an increased risk of developing frailty, an age-related clinical syndrome associated with worse health outcomes. This study examined the effect of self-perception of aging (ie, age discrepancy—individuals feel younger/older than their chronological age and aging satisfaction) on frailty transitions.

**Research Design and Methods:**

We use longitudinal data from 549 HIV−/499 HIV+ sexual minority men aged 50 years or older enrolled in the Multicenter AIDS Cohort Study. To test the association of self-perception of aging on transitions between states of frailty (nonfrail/frail), defined using Fried Frailty Phenotype, a multinomial modeling was used.

**Results:**

With remaining nonfrail as the referent group, participants reporting low aging satisfaction (vs moderate aging satisfaction) had increased odds of transitioning from nonfrail to frail (odds ratio [OR]: 2.72; 95% confidence interval [CI]: 1.56–4.74), frail to nonfrail (OR: 3.40; 95% CI: 1.62–7.12), or remaining frail (frail to frail; OR: 6.64; 95% CI: 3.88–11.38). Participants reporting older subjective age (vs no age discrepancy) had increased odds of transitioning from nonfrail to frail (OR: 2.50; 95% CI: 1.11–5.64), frail to nonfrail (OR: 4.47; 95% CI: 1.85–10.81), or remaining frail (frail to frail; OR: 5.68; 95% CI: 3.06–10.56). High aging satisfaction and younger subjective age were not statistically associated with frailty transitions.

**Discussion and Implications:**

Our findings show that negative self-perception of aging (ie, older subjective age and low aging satisfaction) is associated with frailty transitions (nonfrail to frail, frail to nonfrail, and frail to frail) when compared to remaining nonfrail.

**Translational Significance:** Frailty is associated with an increased risk of adverse health outcomes and poor quality of life. Independent of HIV status, negative self-perception of aging (ie, low aging satisfaction and older subjective age) is associated with frailty transitions among a cohort of sexual minority men aged 50 years or older. Given functional limitations are a core component of the definition of frailty, promoting efforts that would mitigate the impact of negative self-perceptions of aging on functional limitations is important. The impact of this individual-level factor will intersect with other dyadic, community, and structural factors, all of which foster healthy aging.

## Background and Objectives

Over 50% of HIV-positive (HIV+) individuals in the United States are aged 50 or older, and this proportion is expected to increase (1,2). As people living with HIV (PLWH) age, they have an increased risk for the development of age-related comorbidities and geriatric issues such as frailty (3–7). Frailty is an age-related clinical syndrome and a risk factor associated with loss of physical function, hospitalizations, institutionalizations, and death (3,6,8,9). Given these risks, it is critical to investigate the factors driving frailty among older adults.

Frailty phenotype is characterized by the presence of 3 or more of the following components: shrinking (unintentional weight loss), weakness (grip strength), poor endurance and energy, slowness, and low physical activity level (10). In the general population, the estimates about the prevalence of frailty range between 6.9% and 19.5% (10,11). As in the general population, the prevalence of frailty among HIV-positive adults increases with age. The prevalence of frailty among HIV+ adults varies from 5% to 20% (8). Althoff et al. (3) found a 12% prevalence of positive frailty phenotype (FP+) among middle-aged and older male PLWH in the Multicenter AIDS Cohort Study (MACS). This prevalence of FP+ was higher among older PLWH (ie, aged 50–64 years) when compared to HIV-negative (HIV−) men of the same age (3). Additionally, several studies suggest that PLWH are becoming frail at earlier ages when compared to HIV− individuals (3–5,8).

Frailty is a dynamic process; people can transition from a frail state to a prefrail or nonfrail state (3,12). This process can be explained by multiple factors such as differences in the aging process, social and environmental exposures that affect the ability of the systems to respond to stressors (6,13,14). Angulo et al. (13) showed that physical activity and exercise can preserve or improve physical function by reducing inflammation and age-related oxidative damage. On the other hand, it is well documented that inflammation (6,15), age-related comorbidities, and social and behavioral factors such as alcohol use and depressive symptoms are associated with progression to frailty (12,16,17). However, other factors such as self-perceptions of aging are less frequently examined.

Self-perception of aging is the evaluation we hold toward our own aging process, and it is described by 2 components: age discrepancy and aging satisfaction (18–21). The perception about how old people perceive themselves to be in comparison with their chronological age refers to age discrepancy (19,21,22), and the self-assessment of an individual’s own aging process refers to aging satisfaction (19,21).

The experience of aging is different from one individual to another, and this experience can be influenced by the perceptions of aging (23–25). Additionally, some authors consider that the aging process is part of a social construct, and that people can have an age identity different from their chronological age (23,25). As described in the age stereotype embodiment framework, age stereotypes can influence the evaluation we hold toward our own aging process and can be associated with negative self-perception of aging among older adults and worse health outcomes (20,23,25). These internalized beliefs appear to exert influence in the psychological, behavioral, and physiological pathways (23,25).

Several factors such as the perception of good health, high levels of life satisfaction, lower level of perceived disability, and longevity (19,20,22,26) are associated with positive self-perception of aging (ie, high aging satisfaction and younger subjective age). Additionally, younger subjective age has been associated with improved cognitive and physical functioning and reduced depressive symptoms (23). Whereas negative self-perception of aging (ie, low aging satisfaction and older subjective age) is associated with the perception of worse health, the prediction of frailty, functional limitations, and disability (19,23,25–28). Luo and Li (20) found that negative self-perception of aging can affect an individual’s health trajectory. Their models showed that with each one-unit increase in negative self-perception of aging the odds of belonging to the accelerated aging group (26%), characterized by initial worst health status and a fast rate of decline in all health domain, depressed group (17%), characterized by similar initial health status and rates of decline in all health domains than the usual aging group but higher level of depressive symptoms, and usual aging group (9%), characterized by being in the middle in initial health status and rates of decline in all health domains but lower levels of depression, increase when compared with a healthy aging group, characterized by an initial best health status and a slow pace of decline in each health domain (20).

Frailty has a multidimensional impact on the lives of older adults, and it is associated with worse health outcomes. Therefore, it is important to understand how self-perception of aging, an important predictor of quality of life (29), could contribute to frailty states. The aim of this study is to conduct a longitudinal study of HIV+ and HIV− sexual minority men (SMM) enrolled in the MACS from 2016 to 2019 to determine the effect of self-perception of aging on frailty using the stereotype embodiment theory as a framework. We hypothesize that lower aging satisfaction and older subjective age will be independently associated with changes in frailty, after controlling for demographic characteristics, non-HIV-defining comorbidities (ie, hepatitis C, high blood pressure, diabetes, depressive symptoms, dyslipidemia, and kidney disease), and baseline frailty status.

## Research Design and Methods

### Study Design and Population

The MACS is a longitudinal cohort to study the natural and treated history of HIV/AIDS and is comprised of HIV-positive and -negative SMM 4 US sites: Baltimore, Maryland/Washington, DC; Chicago, Illinois; Los Angeles, California; and Pittsburgh, Pennsylvania/Columbus, Ohio. Since 1984, 7 352 participants have been enrolled in the study over the following time periods: 4 954 in 1984–1985, 668 in 1987–1991, 1 350 in 2001–2003, and 380 in 2010–2019. Participants attended semiannual clinic visits where medical history data and specimens are collected using an Audio Computer-Assisted Self-Interview and a standardized clinical examination. Details on the MACS study design have been described elsewhere (30,31). Questionnaires are available at www.aidscohortstudy.org. John Hopkins University Institutional review board (IRB00219740) approved the MACS protocol and informed consent was obtained from all study participants. In this analysis, we included 1 048 (549 HIV−/499 HIV+) men aged 50 years or older who had complete information about age discrepancy and aging satisfaction at Visits 62 (October 2014–March 2015) or 63 (April 2015–September 2015).

#### Time period

Baseline measurement was assessed at either Visit 62 (October 2014–March 2015) or Visit 63 (April 2015–September 2015), wherever first available. Visit 64 (October 2015–March 2016) was the first time point. Visit 67 (April 2017–September 2017) was the mid-point for comorbidity assessment. Visit 70 (October 2018–March 2019) was the second time point.

### Measures

#### Outcome

##### Frailty.

The definition for frailty was adopted within the MACS in 2008. Using Fried’s approach of using the 20th percentile of the cohort of community-dwelling older adults at baseline to set the thresholds for weakness (adjusted for body mass index and sex) and slowness (adjusted for height and sex), the MACS applied the same distributional thresholds to calculate cut-points from its sample at baseline limited to those without HIV. Althoff et al. (3), on whose work our analytic framework is based, noted in their discussion that frailty is more prevalent among HIV− men in the MACS than among the population in the work of Fried et al. (10). Frailty (nonfrail/frail), defined using Fried Frailty Phenotype (10), is the presence of 3 or more of the following: (a) weakness (grip strength measured using a dynamometer is less than 20th percentile of HIV− participants); (b) slowness (timed walk of 4 m that is more than 80th percentile of HIV− men); (c) unintentional weight loss (an affirmative response, “yes,” to the question: “Since your last visit, have you had unintentional weight loss of at least 10 pounds?”); (d) exhaustion (an affirmative response, “yes,” to the question: “During the past 4 weeks, as a result of your physical health, have you had difficulty performing your work or other activities [for example, it took extra effort]?”); and (e) low physical activity (an affirmative response, “Yes, limited a lot,” to the question: “Does your health now limit you in vigorous activities, such as running, lifting heavy objects, participating in strenuous sports?”). Participants with 2 or fewer of the aforementioned criteria were categorized as nonfrail. It was assessed at Visits 64 and 70 for the transition analysis. Additionally, we assessed frailty at Visits 62 or 63 for baseline adjustment in the multinomial model (referred to as baseline frailty status). We also created a second frailty variable, which included a prefrail category, assessed at Visits 64 and 70. It was defined as the following: (a) nonfrail was defined as having none of the above-mentioned criteria, (b) prefrail was defined as having 1 or 2 of the criteria, and (c) frail was defined as having 3 or more of the criteria.

#### Primary predictors

##### *Self-perception of aging*.

Self-perception of aging was comprised of age discrepancy and aging satisfaction and was assessed at Visits 62 or 63. Age discrepancy was calculated as the difference between subjective age (“What age (years) do you feel most of the time?”) and chronological age. Age discrepancy was categorized into 3 categories: older subjective age (subjective age > chronological age), no age discrepancy (subjective age = chronological age), and younger subjective (subjective age < chronological age) (22,24,32). Aging satisfaction was assessed using the Attitudes Towards Aging subscale from the validated Philadelphia Geriatric Center Morale Scale (33–35). The subscale included 5 items: (a) “Things keep getting worse as I get older (Yes/No)”; (b) “I have as much pep as I had over the past 6 months (Yes/No)”; (c) “As I get older, I am less useful (Yes/No)”; (d) “I am as happy now as I was when I was younger (Yes/No)”; and (e) “As I get older, things are . . . than I thought they would be (Better/Worse).” “Yes”/“better” responses were assigned a value of 2 and “no”/“worse” responses were assigned a value of 1. Items 1 and 3 were reverse-coded. All items were summed to obtain a score that ranged from 5 to 10. The resulting values were then categorized into low (5–6), moderate aging satisfaction (7), and high aging satisfaction (8–10) (33).

#### Covariates

##### *HIV status and HIV-related factors*.

HIV status (HIV+/HIV−) was assessed using enzyme-linked immunosorbent assay with a confirmatory Western blot on all MACS participants at their initial visit and at every visit for men who were HIV− at the previous visit. HIV+ participants included all participants who were identified as such at their initial visit and those who seroconverted during study observation. Information regarding plasma HIV RNA levels (viral load, copies/mL) was obtained. Viral load was dichotomized into detectable (>20 copies/mL) and undetectable (≤20 copies/mL).

##### *Age*.

Participants’ chronological age at the visit was calculated from the self-reported date of birth and date of visit.

##### *Race/ethnicity*.

Race was categorized as White, non-Hispanic, Black, non-Hispanic, and Hispanic (ie, White and Black Hispanics were grouped together into a single Hispanic category). Other racial and ethnic categories were removed from the analysis due to small sample sizes.

##### *Education*.

Education was categorized as less than a high school diploma and high school diploma, college, and graduate school.

##### *Comorbidities*.

Comorbidities were measured at Visits 64 and 67 to adjust for the presence of comorbidities at the first frailty assessment and mid-way between the first and second frailty assessments. It included high blood pressure (systolic blood pressure ≥140 mm Hg or diastolic blood pressure ≥90 mm Hg), diabetes (fasting glucose ≥126 mg/dL), liver disease (serum glutamic pyruvic transaminase or serum glutamic oxaloacetic transaminase >150 U/L), kidney disease (estimated glomerular filtration rate <60 mL/min/1.73 m^2^ or urine protein-to-creatinine ratio ≥200), and dyslipidemia (total cholesterol ≥200 mg/dL or low-density lipoprotein cholesterol ≥130 mg/dL or high-density lipoprotein cholesterol <40 mg/dL or triglycerides ≥150 mg/dL) (3). Depressive symptoms were defined using the Center for Epidemiologic Studies—Depression scale, with scores greater than or equal to 16 indicating the presence of significant depressive symptoms (36). Liver and kidney diseases were collapsed into a single indicator variable (liver or kidney disease/no liver or kidney disease). Participants were classified as having a hepatitis C infection if they seroconverted or had an acute infection or chronic infection at Visit 64 only.

### Statistical Methods

**Figure 1.**
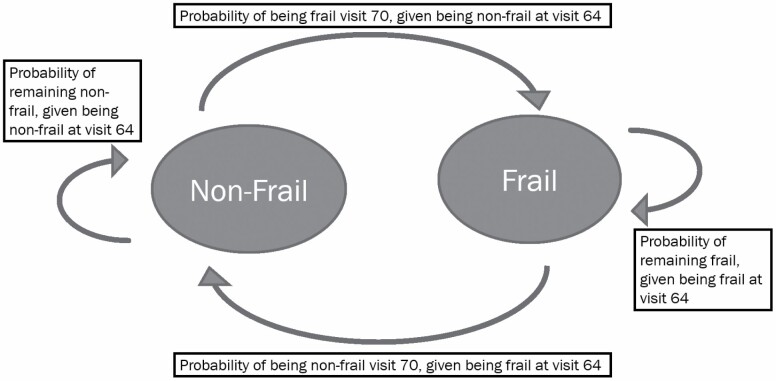
Manifest Markov chain model and transition probabilities.

**Figure 2.**
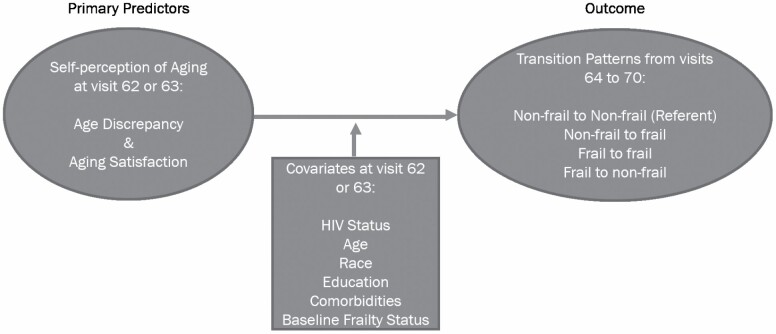
Multinomial model of transition patterns. *Note:* HCV = hepatitis C virus.

Descriptive statistics on the outcomes, primary predictors, and covariates were summarized by HIV status. Differences in variables were tested by chi-square test and Wilcoxon rank-sum test for categorical and continuous variables, respectively. To examine the association of aging satisfaction and age discrepancy on frailty status changes (transitions), we first assessed frailty status (nonfrail/frail) at Visits 64 and 70. Using a manifest Markov chain model, we generated the probability of frailty status changes (transition probabilities), which is defined as the probability of frailty status (nonfrail or frail) at Visit 70, given the frailty status at Visit 64 (Figure 1). Next, a multinomial logistic regression model, which predicts the probability of categorical membership of an outcome, was used to test the association of aging discrepancy and aging satisfaction on the frailty status from Visit 64 to 70 (transition patterns), adjusting for age, HIV status, education, race/ethnicity, baseline frailty (Visit 62 or 63), hepatitis C virus (HCV; at Visit 64), and other comorbidities reported at Visits 64 and 67 (Figure 2). There are 4 mutually exclusive transition patterns: (a) nonfrail to nonfrail (remaining nonfrail), (b) nonfrail to frail, (c) frail to nonfrail, and (d) frail to frail (remaining frail). Descriptive statistics were generated on the transition patterns and presented in Supplementary Table 1. Analyses were also stratified by HIV status to assess possible differences. In post hoc analyses, we also assessed transitions of the second frailty variable that included the prefrail state (selection of 1 or 2 of the Fried criteria). However, the inclusion of the prefrail state created 9 mutually exclusive transition patterns. Some of these patterns had very few participants (as small as *n* = 7) and thus the transition model using these 9 patterns failed to converge. Transition probabilities and adjusted odds ratios (ORs; 95% confidence intervals [CIs]) were reported. Data were analyzed using MPLUS version 8.4 (Muthén & Muthén) and SAS version 9.4 (SAS Institute).

## Results

### Descriptive Statistics

There were 1 048 participants (549 HIV−/499 HIV+) included in the analysis. Participants were majority White, non-Hispanic (73.1%), college-educated (86.8%), reported high aging satisfaction (43.0%), and younger subjective age (71.9%), with a median age of 61 years (interquartile range: 56–66). At baseline, reported comorbidities were HCV (6.1%), hypertension (24.6%), diabetes (13.0%), depressive symptoms (15.4%), dyslipidemia (69.3%), and kidney/liver disease (18.0%). At baseline, 10.9% of participants were frail (Table 1). Among HIV+ participants, 74.6% were undetectable at baseline. Further details by HIV status are given in Table 1.

**Table 1.** Population Characteristics by HIV Status

Variables	HIV-Negative (*n* = 549)	HIV-Positive (*n* = 499)	p	Overall (*N* = 1 048)
Age (years), median (interquartile range)	63 (58–68)	60 (55–64)	<.0001	61 (56–66)
Race/ethnicity, *n* (%)				
Black, non-Hispanic	84 (15.3%)	133 (26.7%)	<.0001	217
Hispanic	22 (4.0%)	43 (8.6%)		65
White, non-Hispanic	443 (80.7%)	323 (64.7%)		766
Education, *n* (%)				
Less than high school	12 (2.2%)	26 (5.2%)	.0002	38
High school	46 (8.6%)	53 (10.6%)		100
College	252 (45.9%)	267 (53.5%)		519
Graduate school	238 (43.4%)	153 (30.7%)		391
Self-perceptions of aging				
Aging satisfaction, *n* (%)				
Low	127 (23.1%)	147 (29.5%)	.0158	274
High	245 (44.6%)	206 (41.3%)		451
Moderate	138 (25.1%)	109 (21.8%)		247
Missing	39 (7.1%)	37 (7.4%)		76
Aging discrepancy, *n* (%)				
Older subjective age	32 (5.8%)	49 (9.8%)	.0088	81
Younger subjective age	416 (75.8%)	338 (67.7%)		754
No age discrepancy	62 (11.3%)	75 (15.0%)		137
Missing	39 (7.1%)	37 (7.4%)		76
Frailty at Visit 62 or 63 (baseline), *n* (%)				
Nonfrail	484 (88.2%)	419 (84.0%)	.3238	903
Frail	49 (8.9%)	65 (13.0%)		114
Missing	16 (2.9%)	15 (3.0%)		31
Comorbidities at Visit 64, *n* (%)				
Hepatitis C	23 (4.2%)	41 (8.2%)	<.0001	64
High blood pressure	137 (25.0%)	121 (24.3%)	.1016	258
Diabetes	63 (11.5%)	73 (14.6%)	.0193	136
Depressive symptoms	79 (14.4%)	82 (16.4%)	.0002	161
Dyslipidemia	371 (67.6%)	355 (71.1%)	<.0001	726
Kidney or liver disease	49 (8.9%)	140 (28.1%)	.0005	189
Comorbidities at Visit 67, *n* (%)				
High blood pressure	124 (22.7%)	97 (20.3%)	.3481	221
Diabetes	68 (13.5%)	78 (18.2%)	.0491	146
Depressive symptoms	98 (19.3%)	94 (21.3%)	.4491	192
Dyslipidemia	387 (77.4%)	356 (82.8%)	.0409	743
Kidney or liver disease	50 (10.0%)	120 (28.1%)	<.0001	170
Viral load detection at Visit 64, *n* (%)				
Undetectable	—	446 (74.6%)	—	446 (74.6%)
Detectable	—	81 (13.6%)		81 (13.6%)
Missing	—	71 (11.9%)		71 (11.9%)
Viral load detection at Visit 70, *n* (%)				
Undetectable	—	362 (68.4%)	—	362 (68.4%)
Detectable	—	96 (18.2%)		96 (18.2%)
Missing	—	71 (13.4%)		71 (13.4%)
Frailty at Visit 64, *n* (%)				
Frail	51 (9.3%)	44 (8.8%)	.1187	95
Nonfrail	428 (78.0%)	391 (78.4%)		819
Missing	70 (12.8%)	64 (12.8%)		134
Frailty at Visit 70, *n* (%)				
Frail	71 (12.9%)	71 (14.2%)	.1927	142
Nonfrail	478 (87.1%)	428 (85.8%)		906
Missing	0 (0.0%)	0 (0.0%)		0

### Frailty Transition Patterns and Probabilities

Overall, there were 4 mutually exclusive frailty transition patterns: (a) 84.3% (*n* = 883) remained nonfrail to nonfrail (NF to NF), (b) 6.2% (*n* = 65) transitioned from nonfrail to frail (NF to F), (c) 6.4% (*n* = 67) transitioned from frail to nonfrail (F to NF), and (d) 3.1% (*n* = 33) remained frail (F to F). The reported transition probabilities were (a) NF to NF: 0.928, (b) NF to F: 0.072, (c) F to NF: 0.378, and (d) F to F: 0.622. Transition probabilities can be interpreted as the probability of the frailty status at Visit 70, given the frailty status Visit 64 (Table 2). The transition probabilities by HIV status are reported in Table 2.

**Table 2.** Transition Probabilities Overall and by HIV Status

Transition Probabilities	Nonfrail at Visit 70	Frail at Visit 70
*Overall*		
Nonfrail at Visit 64	0.928	0.072
Frail at Visit 64	0.378	0.622
*HIV-positive participants*		
Nonfrail at Visit 64	0.930	0.070
Frail at Visit 64	0.333	0.667
*HIV-negative participants*		
Nonfrail at Visit 64	0.916	0.084
Frail at Visit 64	0.363	0.637

### Factors Associated With Frailty Transitions Patterns

#### Aging discrepancy, aging satisfaction, and frailty transition patterns

The multinomial modeling of frailty transition patterns used NF to NF (remaining nonfrail) as the outcome referent group for the result interpretations below. Participants reporting low aging satisfaction (vs moderate aging satisfaction) had increased odds of transitioning from NF to F (OR: 2.72; 95% CI: 1.56–4.74), F to NF (OR: 3.40; 95% CI: 1.62–7.12), or remaining frail (F to F; OR: 6.64; 95% CI: 3.88–11.38). Participants reporting older subjective age (vs no age discrepancy) had increased odds of transitioning from NF to F (OR: 2.50; 95% CI: 1.11–5.64), F to NF (OR: 4.47; 95% CI: 1.85–10.81), or remaining frail (F to F; OR: 5.68; 95% CI: 3.06–10.56). High aging satisfaction and younger subjective age were not statistically associated with frailty status transitions or remaining frail, relative to those with moderate aging satisfaction and no aging discrepancy, respectively (Table 3).

**Table 3.** Overall Multinomial Model of Transition Pattern, Odds Ratio, and 95% Confidence Interval*

Variables	Nonfrail to Frail (referent: Nonfrail to Nonfrail)	Frail to Nonfrail (referent: Nonfrail to Nonfrail)	Frail to Frail (referent: Nonfrail to Nonfrail)
HIV status			
HIV-positive	0.95 (0.55–1.61)	0.94 (0.48–1.85)	1.29 (0.78–2.11)
HIV-negative	Referent	Referent	Referent
Race/ethnicity			
Black, non-Hispanic	1.63 (0.88–3.03)	1.84 (0.79–4.25)	2.48 (1.45–4.24)*
Hispanic	1.78 (1.00–3.17)*	1.60 (0.71–3.58)	1.96 (1.16–3.32)*
White, non-Hispanic	Referent	Referent	Referent
Education			
Less than high school	3.90 (1.53–9.96)*	0.92 (0.11–7.44)	2.44 (0.84–7.05)
High school	2.82 (1.50–5.31)*	1.56 (0.58–4.21)	2.68 (1.48–4.89)*
College	0.96 (0.55–1.66)	1.12 (0.51–2.46)	1.29 (0.76–2.20)
Graduate school	Referent	Referent	Referent
Aging satisfaction			
Low	2.72 (1.56–4.74)*	3.4 (1.62–7.12)*	6.64 (3.88–11.38)*
High	1.07 (0.58–1.98)	0.87 (0.40–1.87)	1.09 (0.61–1.93)
Moderate	Referent	Referent	Referent
Aging discrepancy			
Older subjective age	2.50 (1.11–5.64)*	4.47 (1.85–10.81)*	5.68 (3.06–10.56)*
Younger subjective age	0.89 (0.42–1.90)	0.34 (0.16–0.73)	0.78 (0.4–1.51)
No age discrepancy	Referent	Referent	Referent
Baseline frailty			
Frail	5.29 (2.58–10.87)*	12.2 (5.75–25.64)*	29.41 (16.13–52.63)*
Nonfrail	Referent	Referent	Referent
Comorbidity reported at Visit 64			
Hepatitis C virus (vs None)	3.82 (1.82–8.00)*	2.89 (0.93–8.93)	1.9 (0.78–4.65)
High blood pressure (vs None)	1.73 (0.98–3.04)	0.83 (0.36–1.90)	1.59 (0.92–2.75)
Diabetes (vs None)	1.39 (0.65–2.94)	3.09 (1.24–7.69)*	3.28 (1.79–5.99)*
Depressive symptoms (vs None)	2.03 (1.04–3.97)*	2.86 (1.31–6.25)*	4.67 (2.70–8.06)*
Dyslipidemia (vs None)	0.68 (0.36–1.31)	0.94 (0.38–2.35)	2.29 (0.89–5.92)
Kidney or liver disease (vs None)	1.2 (0.61–2.39)	2.21 (1.01–4.83)*	2.16 (1.22–3.82)*
Comorbidity reported at Visit 67			
High blood pressure (vs None)	1.16 (0.58–2.30)	2.64 (1.12–6.21)*	4.90 (2.86–8.40)*
Diabetes (vs None)	1.27 (0.54–3.01)	1.14 (0.51–2.58)	1.07 (0.60–1.89)
Depressive symptoms (vs None)	4.10 (2.24–7.52)*	2.28 (1.00–5.15)	2.80 (1.51–5.18)
Dyslipidemia (vs None)	0.69 (0.34–1.43)	0.79 (0.26–2.38)	1.20 (0.53–2.70)
Kidney or liver disease (vs None)	1.16 (0.53–2.53)	2.03 (0.88–4.72)	1.98 (1.08–3.61)*

*Adjusted for age.

*p < .05.

#### Covariates and frailty transition patterns

Compared to White, non-Hispanic men, Black, non-Hispanic (OR: 2.48; 95% CI: 1.45–4.27) and Hispanic (OR: 1.96; 95% CI: 1.16–3.32) men had higher odds of remaining frail. Lower education attainment was positively associated with transitions from NF to F (less than high school vs graduate: OR: 3.90; 95% CI: 1.53–9.96; high school vs graduate: OR: 2.82; 95% CI: 1.50–5.31) and remaining frail (high school vs graduate: OR: 2.68; 95% CI: 1.48–4.89). For comorbidities reported at Visit 64, participants with HCV had higher odds of transitioning from NF to F (OR: 3.82; 95% CI: 1.82–8.00). Participants with diabetes had higher odds of transitioning from F to NF (OR: 3.09; 95% CI: 1.24–7.69) or remaining frail (OR: 3.28; 95% CI: 1.79–5.99). Participants with depressive symptoms had higher odds of transitioning from NF to F (OR: 2.03; 95% CI: 1.04–3.97), F to NF (OR: 2.86; 95% CI: 1.31–6.25), or remaining frail (OR: 4.67; 95% CI: 2.70–8.06). Participants reporting kidney or liver disease had higher odds of transitioning from F to NF (OR: 2.21; 95% CI: 1.01–4.83) or remain frail (OR: 2.16; 95% CI: 1.22–3.82). For comorbidities reported at Visit 67, participants reporting depressive symptoms had higher odds of transitioning from NF to F (OR: 4.10; 95% CI: 2.24–7.52) or remain frail (OR: 2.80; 95% CI: 1.51–5.18). Participants reporting kidney or liver disease had higher odds of remaining frail (OR: 1.98; 95% CI: 1.08–3.61). HIV status was not associated with transitioning from NF to F, F to NF, or remaining frail. Further details are reported in Table 3.

When stratified by HIV status controlling by other covariates, results (Tables 4 and 5) were very similar to the overall multinomial model results (Table 3). HIV+ participants reporting low aging satisfaction (vs moderate aging satisfaction) had higher odds of remaining frail (F to F; OR: 5.81; 95% CI: 2.15–15.73), while HIV− participants had higher odds of transitioning from NF to F (OR: 3.24; 95% CI: 1.50–7.03), F to NF (OR: 3.90; 95% CI: 1.43–10.60), or remaining frail (F to F; OR: 7.47; 95% CI: 3.31–16.85). High aging satisfaction and younger subjective age were not statistically associated with frailty status transition or remaining frail, regardless of HIV status. HIV+ participants reporting older subjective age (vs no age discrepancy) had higher odds of transitioning from NF to F (OR: 3.16; 95% CI: 1.11–9.05), F to NF (OR: 7.06; 95% CI: 2.26–22.05), or remaining frail (F to F; OR: 5.81; 95% CI: 2.15–15.73), while HIV− participants had increased odds of remaining frail (OR: 8.27; 95% CI: 3.08–22.21). Further details are reported in Tables 4 and 5.

**Table 4.** HIV-Positive Only Multinomial Model of Transition Pattern, Odds Ratio, and 95% Confidence Interval*

Variables	Nonfrail to Frail (referent: Nonfrail to Nonfrail)	Frail to Nonfrail (referent: Nonfrail to Nonfrail)	Frail to Frail (referent: Nonfrail to Nonfrail)
Race/ethnicity			
Black, non-Hispanic	1.80 (0.56–5.75)	0.93 (0.29–2.99)	2.29 (1.00–5.29)
Hispanic	2.15 (0.63–7.28)	0.81 (0.27–2.41)	1.97 (0.82–4.70)
White, non-Hispanic			
Education			
Less than high school	6.02 (1.37–26.39)	1.66 (0.21–13.40)	1.53 (0.14–16.65)
High school	3.52 (1.24–9.96)	0.79 (0.17–3.60)	0.93 (0.27–3.19)
College	0.87 (0.31–2.44)	0.55 (0.17–1.84)	0.88 (0.39–1.97)
Graduate school			
Aging satisfaction			
Low	2.73 (0.91–8.15)	2.20 (0.79–6.16)	5.81 (2.15–15.73)
High	0.94 (0.37–2.36)	1.03 (0.28–3.89)	1.06 (0.38–3.00)
Moderate			
Aging discrepancy			
Older subjective age	3.16 (1.11–9.05)	7.06 (2.26–22.05)	5.81 (2.15–15.73)
Younger subjective age	0.82 (0.24–2.86)	0.37 (0.11–1.21)	1.06 (0.38–3.00)
No age discrepancy			
Baseline frailty			
Frail	3.75 (0.63–22.22)	7.19 (2.22–23.26)	19.23 (6.45–58.82)
Nonfrail			
Comorbidity reported at Visit 64			
Hepatitis C virus (vs None)	3.66 (1.19–11.24)	1.70 (0.36–8.06)	1.92 (0.54–6.80)
High blood pressure (vs None)	1.84 (0.62–5.43)	5.03 (0.51–50.00)	2.20 (0.73–6.62)
Diabetes (vs None)	0.79 (0.08–7.52)	3.89 (0.95–15.87)	2.99 (0.90–9.90)
Depressive symptoms (vs None)	2.28 (0.81–6.41)	2.72 (0.87–8.47)	3.70 (1.59–8.70)
Dyslipidemia (vs None)	0.59 (0.19–1.82)	No-Est	1.17 (0.30–4.63)
Kidney or liver disease (vs None)	1.81 (0.54–6.13)	6.10 (1.47–25.64)	1.31 (0.47–3.68)
Comorbidity reported at Visit 67			
High blood pressure (vs None)	0.89 (0.14–5.52)	0.70 (0.20–2.53)	0.47 (0.07–3.11)
Diabetes (vs None)	0.56 (0.05–6.58)	5.03 (1.12–22.73)	2.30 (0.73–7.30)
Depressive symptoms (vs None)	2.00 (0.63–6.29)	2.30 (0.66–8.06)	3.57 (1.47–8.70)
Dyslipidemia (vs None)	0.47 (0.12–1.88)	No-Est	0.81 (0.18–3.65)
Kidney or liver disease (vs None)	2.01 (0.51–7.94)	4.81 (1.28–18.18)	1.28 (0.37–4.41)

*Adjusted for age.

**Table 5.** HIV-Negative Only Multinomial Model of Transition Pattern, Odds Ratio, and 95% Confidence Interval*

Variables	Nonfrail to Frail (referent: Nonfrail to Nonfrail)	Frail to Nonfrail (referent: Nonfrail to Nonfrail)	Frail to Frail (referent: Nonfrail to Nonfrail)
Race/ethnicity			
Black, non-Hispanic	1.85 (0.75–4.56)	2.83 (0.86–9.34)	1.53 (0.65–3.62)
Hispanic	1.83 (0.76–4.37)	2.09 (0.64–6.82)	1.25 (0.48–3.24)
White, non-Hispanic			
Education			
Less than high school	5.70 (1.43–22.75)	2.19 (0.06–76.40)	2.57 (0.49–13.52)
High school	2.75 (1.07–7.06)	2.01 (0.46–8.85)	3.43 (1.45–8.10)
College	1.34 (0.60–2.98)	2.74 (0.90–8.31)	1.62 (0.73–3.63)
Graduate school			
Aging satisfaction			
Low	3.24 (1.50–7.03)	3.90 (1.43–10.60)	7.47 (3.31–16.85)
High	1.10 (0.49–2.44)	0.70 (0.26–1.86)	1.15 (0.53–2.51)
Moderate			
Aging discrepancy			
Older subjective age	2.79 (0.81–9.63)	1.06 (0.10–11.75)	8.27 (3.08–22.21)
Younger subjective age	1.10 (0.33–3.65)	0.33 (0.13–0.87)	0.84 (0.30–2.34)
No age discrepancy			
Baseline frailty			
Frail	9.43 (3.61–24.39)	16.95 (5.75–50.00)	37.04 (15.63–90.91)
Nonfrail			
Comorbidity reported at Visit 64			
Hepatitis C virus (vs None)	5.62 (1.36–23.26)	4.20 (0.80–22.22)	1.54 (0.09–27.03)
High blood pressure (vs None)	0.88 (0.39–1.99)	0.83 (0.28–2.48)	1.63 (0.57–4.67)
Diabetes (vs None)	1.78 (0.50–6.37)	0.92 (0.14–5.85)	3.47 (1.44–8.40)
Depressive symptoms (vs None)	2.24 (0.81–6.21)	2.77 (0.86–8.93)	6.90 (3.19–14.71)
Dyslipidemia (vs None)	0.69 (0.26–1.80)	0.38 (0.13–1.14)	3.08 (0.84–11.24)
Kidney or liver disease (vs None)	0.78 (0.09–6.41)	0.57 (0.08–4.03)	2.80 (1.03–7.58)
Comorbidity reported at Visit 67			
High blood pressure (vs None)	0.86 (0.36–2.06)	1.18 (0.42–3.29)	1.67 (0.82–3.39)
Diabetes (vs None)	2.00 (0.71–5.62)	0.94 (0.13–6.80)	4.02 (1.78–9.09)
Depressive symptoms (vs None)	7.30 (3.26–16.13)	4.27 (1.36–13.33)	6.02 (2.86–12.66)
Dyslipidemia (vs None)	0.79 (0.28–2.23)	0.55 (0.18–1.75)	2.39 (0.63–9.01)
Kidney or liver disease (vs None)	0.48 (0.02–11.36)	0.50 (0.06–3.88)	3.01 (1.14–8.00)

*Adjusted for age.

## Discussion and Implications

This longitudinal study provides additional information about the association between self-perception of aging and frailty transitions among HIV-positive and -negative men enrolled in the MACS. Our findings show that negative self-perception of aging (ie, low aging satisfaction and older subjective age) was positively associated with frailty transitions (NF to F or F to NF) or remaining frail (F to F) after controlling for race, education, HIV status, and comorbidities when compared to participants with moderate aging satisfaction and no age discrepancy. Lower educational attainment, Black non-Hispanic or Hispanic, and the presence of comorbidities such as depressive symptoms, Hepatitis C, diabetes, and kidney disease were also positively associated with frailty transitions or remaining frail. Similar statistically significant associations were observed in the multinomial model stratified by HIV status. In both groups, negative self-perception of aging (ie, low aging satisfaction and older subjective age) was associated with all frailty transitions.

These findings are consistent with a previous study that found that negative self-perception of aging is associated with the prediction of frailty (28). Warmoth et al. (28) found that individuals with a negative self-perception of aging were more likely to be frailer after 6 years. Additionally, negative self-perception of aging has also been in association with functional limitations (26–28). Functional limitations are an important component in the definition of frailty and have also been associated with poor health outcomes such as cardiovascular disease and depressive symptoms (7,26,28,32). Levy (25) explained that negative age stereotypes can affect physiological pathways which can negatively influence physical functioning and health outcomes. Additionally, our results regarding covariates were also consistent with other studies, showing that lower education attainment, Black non-Hispanic and Hispanic, and presence of comorbidities were associated with remaining frail (3,12,16,17,23,28).

It would have been expected to find low aging satisfaction and older subjective age to be negatively associated with transitioning from F to NF. However, it should be noted that in the multinomial modeling of the frailty transition patterns, the outcome referent group includes participants who remained nonfrail. The referent level, nonfrail, could be considered the “healthier level” because these individuals have not reported any of the criteria associated with frailty: shrinking (unintentional weight loss), weakness (grip strength), poor endurance and energy, slowness, and low physical activity level. In contrast, the group that transitioned from F to NF has reported some of the criteria used to define the frailty phenotype. A higher proportion of participants who transitioned from F to NF reported low aging satisfaction and older subjective age compared to participants who remained nonfrail (Supplementary Table 1). Additionally, the rate of reported low aging satisfaction and older subjective age among participants who remained frail is higher than those who transitioned from F to NF. When the referent level of the model was changed to be remaining frail, we found that those who went from F to NF were less likely to report older subjective age (OR: 0.577 [95% CI: 0.225–1.479]) and low aging satisfaction (OR: 0.386 [95% CI: 0.182–0.821]), which is what we would expect. Therefore, our findings remain in line with the work of Warmoth et al. (28) about the association between negative self-perception of aging and frailty.

The effect size for transitioning from F to NF was larger than transitioning from NF to F due to more participants transitioning from F to NF (*n* = 67) than those transitioning from NF to F (*n* = 65). We theorized that some participants who are nonfrail may actually be prefrail, explaining the transitions between F and NF. We found that 65.3% of participants who were classified as nonfrail in our analyses can be classified as prefrail. Of the participants who transitioned from F to NF using the original variable (*n* = 67), 60 transitioned from frail to prefrail. Unfortunately, when we included the prefrail state in the model, the model failed to converge because of the small number of participants in some of the categories (F to NF: *n* = 7 and NF to F: *n* = 9).

This study has some limitations. Our analyses were restricted to men enrolled in the MACS and the findings may not be generalizable to other SMM living with and without HIV. Women could not be included in these analyses because MACS does not enroll women. Due to the limited sample size, we were unable to fully examine the prefrail state in the transition model. The follow-up period for the frailty measures was 3 years, due to the availability of the primary predictor (ie, self-perception of aging), which may affect the number of transitions. Longer follow-up times may increase the number of transitions because frailty progresses with age, comorbid conditions, and lifestyle factors (10,13). Despite this limitation, this study includes a large cohort of multiethnic participants and an appropriate comparison group of HIV− men that allow the evaluation of self-perception of aging (ie, aging satisfaction and aging discrepancy) and frailty.

Our results highlight the importance of promoting positive self-perception of aging (ie, aging satisfaction and age discrepancy). Tovel et al. (2019) found that the positive effect of self-perception of aging on physical functioning and that relationship can be mediated by self-efficacy. Other studies indicate that promoting a positive self-perception of aging can facilitate the implementation of self-care models that can prevent or control the progression of comorbidities and frailty (37,38,39). In conclusion, this study provides support about the influence that psychological factors such as self-perception of aging have on frailty transitions.

## Supplementary Material

igab035_suppl_Supplementary_Materials_1
