# Supplementary material for: Negative Perception of Aging Is Associated With Frailty Transitions Within a Cohort of Sexual Minority Men
Source: Innov Aging. 2021 Sep 3;5(4):igab035. doi: 10.1093/geroni/igab035 (PMC8599189; doi:10.1093/geroni/igab035)
Supplement: igab035_suppl_Supplementary_Materials_1 [file igab035_suppl_supplementary_materials_1.docx]

Online Supplementary Table 1. Characteristics of Transition Patterns

| **Variables** | **Non-Frail to Non-Frail** | **Non-Frail to Frail** | **Frail to Non-Frail** | **Frail to Frail** |
| --- | --- | --- | --- | --- |
| **HIV status** |  |  |  |  |
| HIV-positive | 47.3% (44.0%-50.7%) | 45.9% (33.1%-58.8%) | 45.8% (29.6%-62.0%) | 53.6% (41.8%-65.4%) |
| HIV-negative | 52.7% (49.3%-56.0%) | 54.1% (41.2%-66.9%) | 54.2% (38.0%-70.4%) | 46.4% (34.6%-58.2%) |
| **Race/Ethnicity** |  |  |  |  |
| Black, non-Hispanic | 18.4% (15.8%-21.1%) | 26.9% (15.2%-38.6%) | 29.3% (12.7%-45.9%) | 35.9% (24.3%-47.6%) |
| Hispanic | 6.2% (4.6%-7.9%) | 9.9% (2.4%-17.5%) | 5.0% (1.8%-11.7%) | 3.1% (0.0%-7.6%) |
| White, non-Hispanic | 75.3% (72.4%-78.3%) | 63.1% (50.2%-76.1%) | 65.7% (48.2%-83.2%) | 60.9% (48.9%-72.9%) |
| **Education** |  |  |  |  |
| Less than high school | 2.9% (1.8%-4.0%) | 10.4% (2.5%-18.2%) | 2.7% (2.6%-7.9%) | 6.7% (0.6%-12.9%) |
| High school | 8.1% (6.3%-10.0%) | 15.5% (6.2%-24.7%) | 13.5% (1.5%-25.4%) | 18.2% (9.0%-27.3%) |
| College | 51.2% (47.9%-54.6%) | 35.3% (23.0%-47.7%) | 48.8% (32.5%-65.1%) | 43.1% (31.4%-54.7%) |
| Graduate school | 37.8% (34.5%-41.0%) | 38.8% (26.2%-51.4%) | 35.1% (17.8%-52.3%) | 32.0% (20.8%-43.2%) |
| **Aging satisfaction** |  |  |  |  |
| Low | 22.4% (19.4%-25.3%) | 43.9% (30.9%-57.0%) | 49.5% (31.9%-67.0%) | 65.7% (54.2%-77.2%) |
| High | 52.1% (48.6%-55.7%) | 31.8% (19.8%-43.7%) | 22.3% (8.8%-35.8%) | 10.4% (2.8%-18.0%) |
| Moderate | 25.5% (22.4%-28.5%) | 24.3% (13.4%-35.2%) | 28.2% (13.2%-43.3%) | 23.9% (13.9%-34.0%) |
| **Aging Discrepancy** |  |  |  |  |
| Older subjective age | 5.7% (4.0%-7.3%) | 13.1% (4.5%-21.7%) | 21.2% (7.8%-34.7%) | 25.5% (15.3%-35.8%) |
| Younger subjective age | 81.3% (78.5%-84.0%) | 72.5% (61.0%-83.9%) | 48.4% (31.5%-65.3%) | 58.3% (46.6%-70.1%) |
| No age discrepancy | 13.0% (10.7%-15.4%) | 14.4% (5.4%-23.5%) | 30.3% (15.4%-45.3%) | 16.1% (7.6%-24.6%) |
| **Baseline frailty (visit 62 or 63)** |  |  |  |  |
| Non-frail | 95.1% (93.6%-96.6%) | 78.6% (67.9%-89.4%) | 61.6% (46.0%-77.1%) | 40.0% (28.2%-51.8%) |
| Frail | 4.9% (3.4%-6.4%) | 21.4% (10.6%-32.1%) | 38.4% (22.9%-54.0%) | 60.0% (48.2%-71.8%) |
| **Comorbidity reported at visit 64** |  |  |  |  |
| Hepatitis C | 5.1% (3.5%-6.6%) | 16.9% (7.5%-26.3%) | 13.3% (1.1%-25.5%) | 9.2% (2.2%-16.2%) |
| High blood pressure | 24.6% (21.7%-27.6%) | 36.1% (23.6%-48.6%) | 21.4% (7.8%-34.9%) | 34.2% (22.5%-46.0%) |
| Diabetes | 13.4% (10.8%-16.1%) | 17.7% (7.3%-28.2%) | 32.4% (13.8%-51.1%) | 33.7% (21.2%-46.3%) |
| Depressive symptoms | 13.9% (11.5%-16.4%) | 24.8% (12.9%-36.6%) | 31.6% (15.6%-47.6%) | 43.1% (30.6%-55.5%) |
| Dyslipidemia | 80.7% (77.9%-83.6%) | 74.1% (62.1%-86.1%) | 79.7% (65.4%-94.1%) | 90.6% (82.6%-98.5%) |
| Kidney or liver disease | 18.2% (15.5%-21.0%) | 21.2% (10.2%-32.2%) | 33.1% (16.6%-49.6%) | 32.5% (20.7%-44.3%) |
| **Comorbidity reported at visit 67** |  |  |  |  |
| High blood pressure | 21.6% (18.7%-24.4%) | 19.2% (9.0%-29.4%) | 23.9% (9.5%-38.3%) | 22.7% (13.1%-32.2%) |
| Diabetes | 13.3% (10.8%-15.9%) | 16.4% (5.1%-27.6%) | 31.9% (11.0%-52.7%) | 30.1% (17.9%-42.4%) |
| Depressive symptoms | 15.2% (12.6%-17.8%) | 42.4% (28.7%-56.1%) | 32.2% (14.3%-50.1%) | 46.8% (34.3%-59.3%) |
| Dyslipidemia | 80.0% (77.1%-82.9%) | 73.4% (60.0%-86.9%) | 83.5% (68.6%-98.5%) | 82.7% (71.4%-94.1%) |
| Kidney or liver disease | 16.7% (14.0%-19.4%) | 18.9% (7.4%-30.4%) | 29.0% (12.3%-45.7%) | 28.4% (16.7%-40.0%) |
